# Role of CT Imaging With Three-Dimensional Maximum Intensity Projection Reconstruction in the Evaluation of Portal Vein Variants at a Tertiary Care Hospital

**DOI:** 10.7759/cureus.11733

**Published:** 2020-11-28

**Authors:** Muhammad Asad Ullah, Muhammad Saad Ahmed, Kamran Hamid, Muhammad Ali, Muhammad Kashif Shazlee, Jaideep Darira

**Affiliations:** 1 Diagnostic Radiology, Dr. Ziauddin Hospital, Karachi, PAK; 2 Imaging Services, The Indus Hospital, Karachi, PAK

**Keywords:** maximum intensity projection, computed tomography, portal vein variant

## Abstract

Introduction: Portal vein (PV) is the principal blood vessel transporting blood from the alimentary tract and spleen to the liver. The aim of this study is to determine the prevalence of PV anatomical variations in our population using multidetector CT with maximum intensity projection (MIP) technique at a tertiary care hospital.

Methods: This cross-sectional study was prospectively conducted from November 2018 to June 2019 in the Department of Radiology at a tertiary care hospital in Karachi. After informed consent, all the patients with no known hepatic pathology undergoing routine abdomen CT were included in this study. Patients with previous hepatic resection surgeries, undiagnosed large hepatic tumors/metastasis, and those with PV thrombosis were excluded.

Results: A total of 500 patients (256 males and 244 females) were included in the study; the mean age of female patients was relatively higher as compared to the male patients (53.80 ± 18.44 vs. 44.15 ± 19.94 years; p = 0.000). Standard PV anatomy (type 1) was found in 438 patients (87.6%). Trifurcation (type 2) occurred in 18 patients (3.6%). Right posterior portal vein as the first branch of main PV (type 3) was found in 22 patients (4.4%). A separate branch of the right portal vein (RPV) to segment VII (type 4) and separate branch of the RPV to segment VI (type 5) were found in 6 (1.2%) and 16 (3.2%) patients, respectively.

Conclusion: Our study displayed a relatively higher frequency of standard PV anatomy (type 1) compared to previous studies. We highlight the role of MIP in the analysis of hepatic venous anatomy with its utility demonstrating improved detection of variations.

## Introduction

Portal vein (PV) is the principal blood vessel transporting blood from the alimentary tract and spleen to the liver, accountable for 75% of hepatic blood supply [1]. The coalition of splenic and superior mesenteric veins lying posterior to the neck of pancreas leads to the formation of PV. PV is not considered as a true vein due to the fact that it does not directly return blood to the heart [2,3].

Normally, the portal vein splits at porta hepatis into two terminal branches: the right portal vein (RPV) and the left portal vein (LPV) [1]. The larger RPV further bisects into the right anterior portal vein (RAPV) and the right posterior portal vein (RPPV). The RAPV supplies hepatic segments V and VIII while segments VI and VII are served by the RPPV. LPV accounts for the blood supply of the left liver lobe [2-4].

Studies suggest that this classical anatomy is observed in only about 65% to 70% of individuals and disparity in PV anatomy is encountered in a notable number of subjects [2,5]. The trifurcation of the main PV (MPV) is the most common variant. Other variations include RPPV as the foremost ramification from the MPV, a separately originating branch to segment VI from RPV and the separate branch to segment VII from RPV [1-3]. There also exist few other variations but these are very rare.

With the growing utilization of interventional radiological techniques like PV embolization, transjugular intrahepatic portosystemic shunting (TIPS), alongside liver transplantation and liver resections, the accurate knowledge of PV branching anatomy is of great significance for surgeons and interventionists to avoid undesirable complications [2,3,5-7].

Intricate hepatic vasculature necessitates comprehensive pre-operative assessment to avoid procedure-related maladies. That is why the lately developed multidetector CT (MDCT) imaging software and post-processing techniques are of utmost importance especially while planning for liver transplant surgeries or segmental resections [8]. A study suggests that three-dimensional volume rendering (3D-VR) and maximum intensity projection (MIP) techniques depict detailed hepatic vascular anatomy with high precision and veracity [6,8]. Though MIP reconstructed images may cause some ambiguity due to motion artifacts, image overlapping, and encumbrance from tissue calcifications, it is a rapid post-processing technique that enhances vascular details with high accuracy [8].

Though studies have been conducted worldwide to find out different PV anatomical variants, but there is an exiguity of local data in this domain. With rapidly evolving hepatobiliary interventional and surgical procedures in our region, we must identify these variations and their respective prevalence among the local population.

The aim of this study is to determine the prevalence of PV anatomical variants in our population using abdominal MDCT with MIP technique at a tertiary care hospital.

## Materials and methods

Following approval from the Ethical Review Committee, this cross-sectional study was prospectively conducted from November 2018 to June 2019 in the Department of Radiology at a tertiary care hospital in Karachi. After informed consent, all the patients with no known hepatic pathology undergoing routine abdomen CT were included in this study irrespective of age and gender. Patients with previous hepatic resection surgeries, undiagnosed large hepatic tumors/metastatic lesions, and those with PV thrombosis were excluded.

Contrast-enhanced CT of the abdomen was performed using a 16 slice CT scanner machine. The field of view extended from the xiphisternum to the symphysis pubis. Nonionic contrast iopromide (Ultravist 300; Bayer Pharma AG, Germany) having a concentration of 300 mg/ml was given intravenously. An automated power injector was used to deliver contrast via an 18-gauge cannula. The flow rate was 3 ml/s. Images were acquired in the portal venous phase at 60 s. A slice thickness of 1 mm was obtained with a 5-mm reconstruction interval.

Axial images transferred to a workstation (Vitrea) were analysed using post-processing techniques including 3D-VR, multiplanar reconstruction (MPR), and MIP. Each scan was interpreted by a fourth-year postgraduate trainee and two radiologists having experience of five years each; equivocal cases were further evaluated by another radiologist having 20 years of experience in cross-sectional imaging. Data for different PV variants along with demographic details and utility of MIP was recorded. SPSS version 20 (IBM Corp., Armonk, NY) was used for statistical analysis.

## Results

We prospectively interpreted and evaluated PV anatomy in 569 abdominal contrast CT scans. Out of these, 69 scans were omitted with majority (41) due to technical issues like sub-optimal portal venous opacification and imaging artifacts. Other patients excluded from this study had obscuration or distortion of normal portal venous anatomy owing to previously undiagnosed pathologies such as PV thrombosis, cavernous transformation of the PV, hepatocellular carcinoma, cholangiocarcinoma, metastatic deposits, etc.

Therefore, a total of 500 patients (256 males and 244 females) were included in this study. The mean age of female subjects was relatively higher as compared to the males (53.80 ± 18.44 vs. 44.15 ± 19.94 years; p = 0.000).

**Figure 1:**
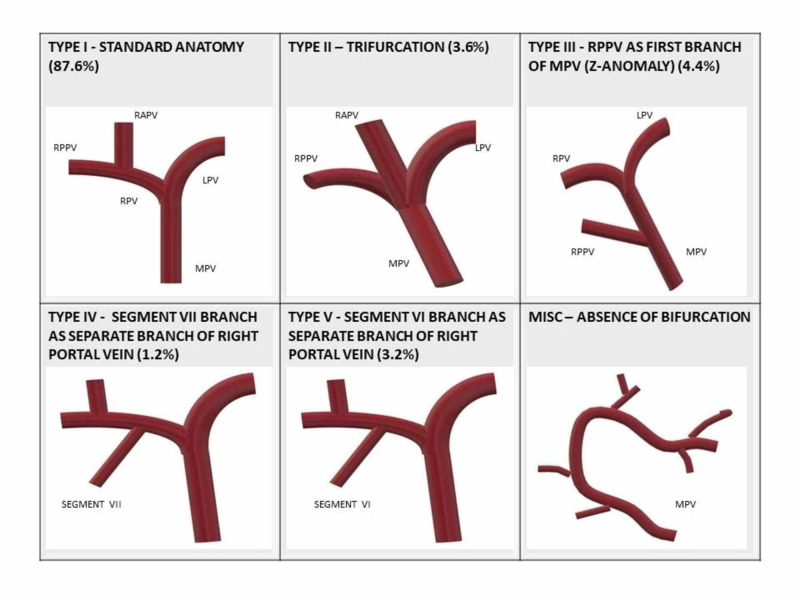
Schematic diagram of different types of variations in the portal vein RPPV, right posterior portal vein; MPV, main portal vein.

Table 1 and Figure 1 show the frequencies of PV variation, where standard PV anatomy was found in most number of patients, i.e., 438 (87.6%). RPPV as the first branch of MPV (type 3) was found in 22 patients (4.4%) and trifurcation (type 2) occurred in 18 patients (3.6%). A separate branch of RPV to segment VII (type 4) and separate branch of RPV to segment VI (type 5) were found in 6 (1.2%) and 16 (3.2%) patients, respectively.

**Table 1:** Frequencies of different types of anatomical variation in PV PV, portal vein. Data is presented as frequencies, n (%).

Type of Variation	Frequencies	Percentage (%)
Type 1 - Standard	438	87.6
Type 2 - Trifurcation	18	3.6
Type 3 - Right posterior portal vein as the first branch of PV	22	4.4
Type 4 - Separate branch of the right portal vein to segment VII	6	1.2
Type 5 - Separate branch of the right portal vein to segment VI	16	3.2
Total	500	100

The number of unconventional branching patterns of portal vein detected with MIP (46) was higher compared to that observed without MIP (16). Utilizing MIP, we identified 16 patients (25.80%) of trifurcation, 14 patients (22.58%) of Z-anomaly, 12 patients (19.35%) of type 5, and 4 patients (6.45%) of type 4 pattern. Although the p-value was found insignificant (p = 0.322), but without MIP diagnostic efficacy was considerably low for identifying the variations (Table 2). Overall, this makes addition of MIP to MDCT images three times more effective in the interpretation of variant anatomy than VR and MPR alone.

**Table 2:** Identification of the type of variation in PV with and without MIP PV, portal vein; MIP, maximum intensity projection. Data is presented as n (%). p < 0.05 is considered as statistically significant. Chi-square tests were applied.

	Variation in PV					
CT software	Trifurcation	Right posterior portal vein as the first branch of PV	Separate branch of the right portal vein to segment VII	Separate branch of the right portal vein to segment VI	Total = 62 (100%)	p-value
With MIP	16 (25.80%)	14 (22.58%)	4 (6.45%)	12 (19.35%)	46 (74.18%)	0.322
Without MIP	2 (3.22%)	8 (12.90%)	2 (3.22%)	4 (6.45%)	16 (25.82%)	

Table 3 shows the gender-wise distribution of variations in PV. In female patients, the percentage of RPPV as the first branch of PV (54.55%) and separate branch of the RPV to segment VI (62.5%) is higher as compared to male patients, whereas type 2 and type 4 patterns were more common in male patients, estimating 55.55% and 66.66%, respectively.

**Table 3:** Distribution of PV variation based on gender PV, portal vein. Data is presented as n (%).

PV Variation	Male	Female	Total
Standard	226 (51.59%)	212 (48.41%)	438
Trifurcation	10 (55.55%)	8 (44.45%)	18
Right posterior portal vein as the first branch of PV	10 (45.45%)	12 (54.55%)	22
Separate branch of the right portal vein to segment VII	4 (66.66%)	2 (33.34%)	6
Separate branch of the right portal vein to segment VI	6 (37.5%)	10 (62.5%)	16

## Discussion

Portal vein is responsible for 75% of the hepatic blood supply. Its tributaries include splenic and superior mesenteric veins that drain blood from the spleen and midgut, respectively. They form a confluence posterior to the neck of pancreas, corresponding to the level of L2 lumbar vertebra, from where the portal vein protracts for a length of 5-8 cm before bifurcating at porta hepatis [2,7,9]. The left portal vein extends medially vascularizing segments II, III, IV, and caudate while the right portal vein further bisects into right anterior portal vein and right posterior portal vein [9,10]. RAPV supplies blood to segments V and VIII while RPPV to segments VI and VII [2,7,9].

Vitelline veins are the embryological predecessors of the portal vein. The right and left vitelline veins compose a mesh encircling duodenum via paired ventral and single dorsal interconnecting vascular channels. Some of these channels revert and others evolve as programmed genetically to generate the conventional portal vein and its branches. Deviations in this scripted evolution leads to morphologically distinct patterns [11,12].

In recent years, there has been an escalation in the tally of hepato-biliary interventions, liver transplants, and partial resections in our region. The complexity of hepatic vasculature, therefore, calls for in-depth analysis where unfamiliarity with commonly unreported portal vein variants may lead to calamitous consequences [13].

**Figure 2:**
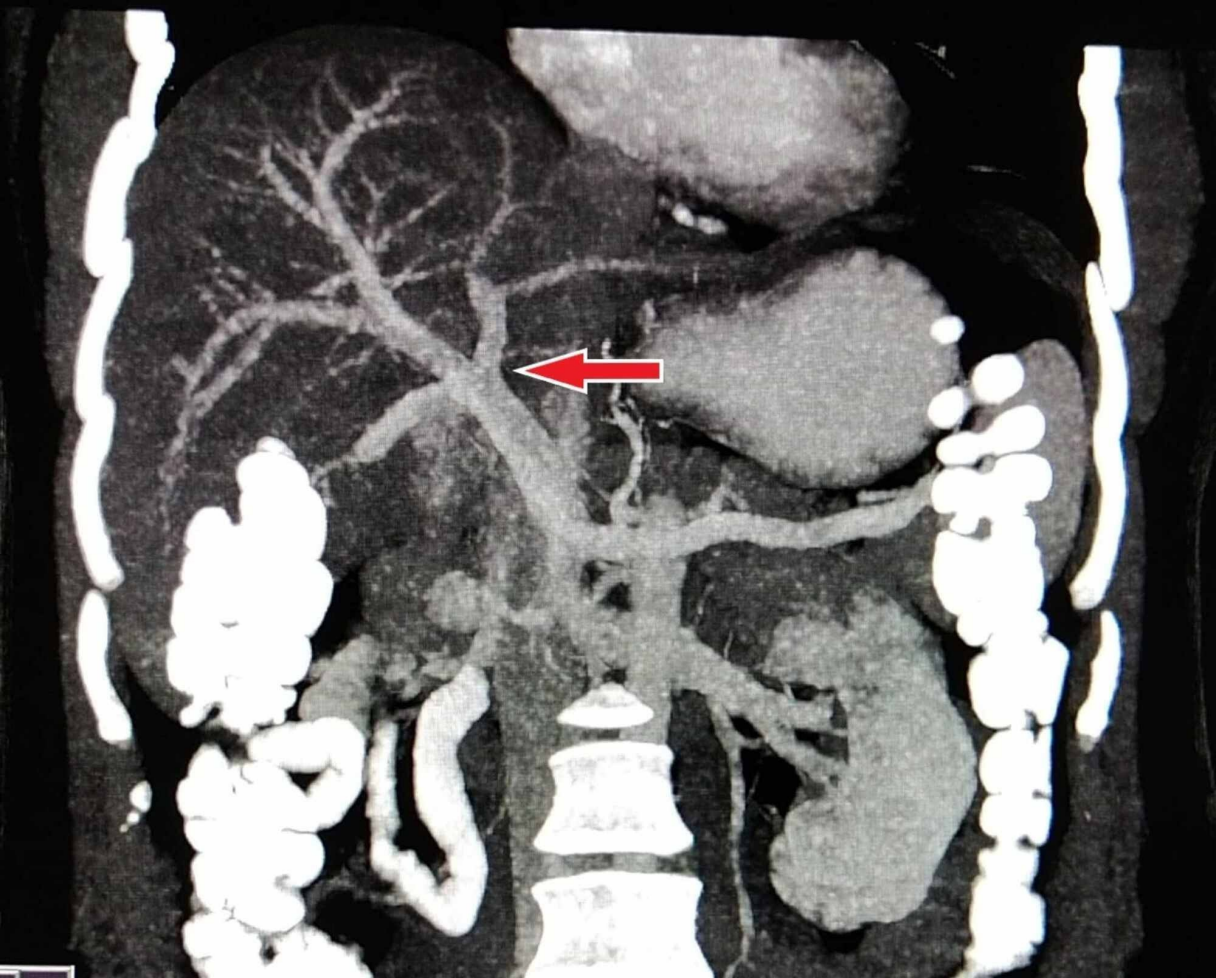
Contrast-enhanced CT MIP reconstructed coronal image showing the trifurcation of the main portal vein (type 2) MIP, maximum intensity projection.

**Figure 3:**
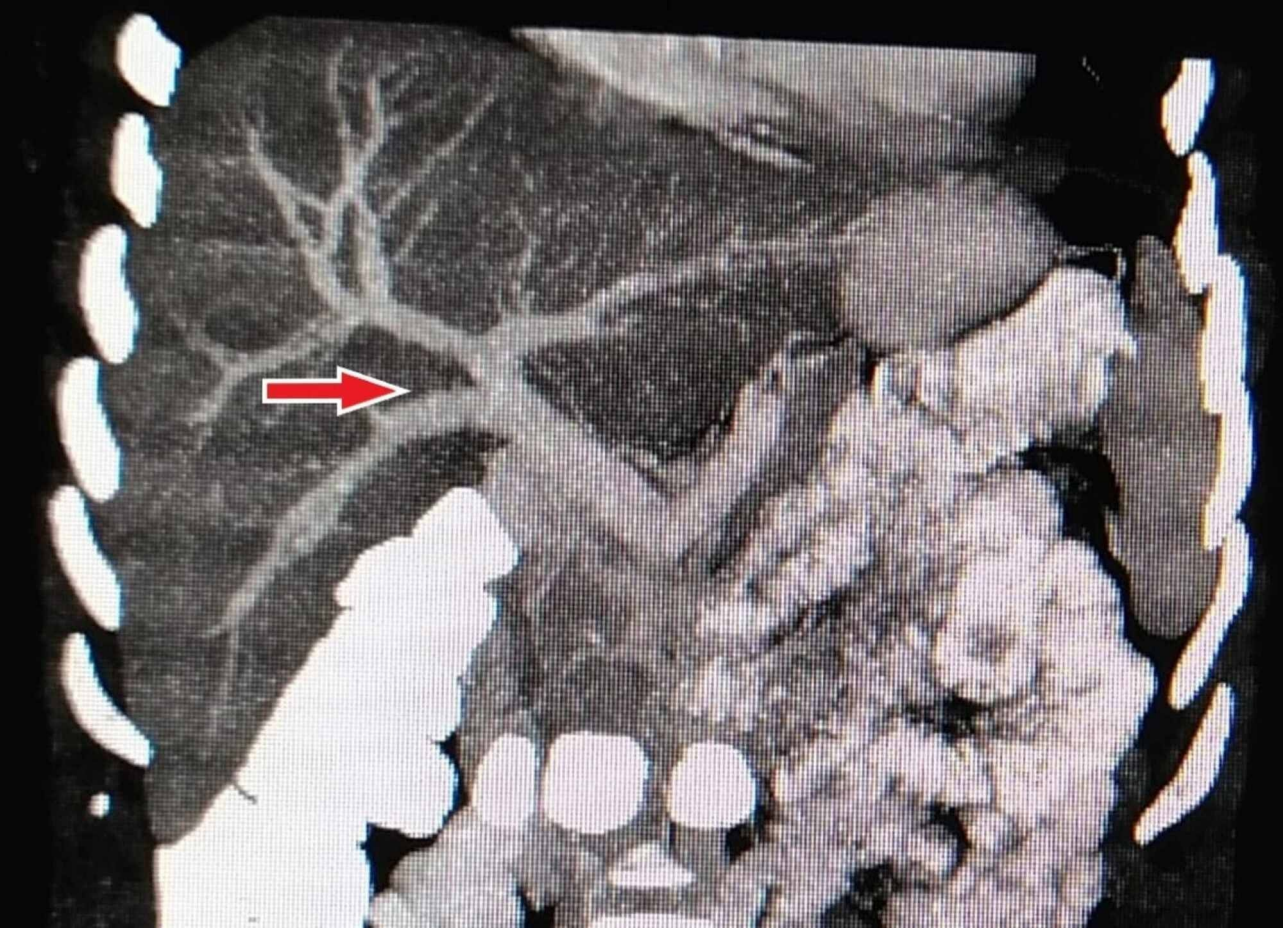
Contrast-enhanced CT MIP reconstructed coronal image showing the origin of RPPV as the first branch of the main portal vein (type 3) MIP, maximum intensity projection; RPPV, right posterior portal vein.

The percentage of the conventional branching pattern of portal vein was higher (87.6%) in our study compared to previous studies with deviation from standard anatomy only observed in 12.4% patients. Saylisoy et al. [14] and Singh et al. [15] reported similar proportions of normal anatomy, i.e., 88% and 89%, respectively. PV trifurcation (type 2) was observed in 18 (3.6%) patients (Figure 2). Our study demonstrated type 3 - ‘Z’ anomaly (right posterior portal vein as the first branch of the MPV) as the most frequently encountered anatomical variation of portal vein (Figure 3), which is in concordance with Covey et al. [13].

**Figure 4:**
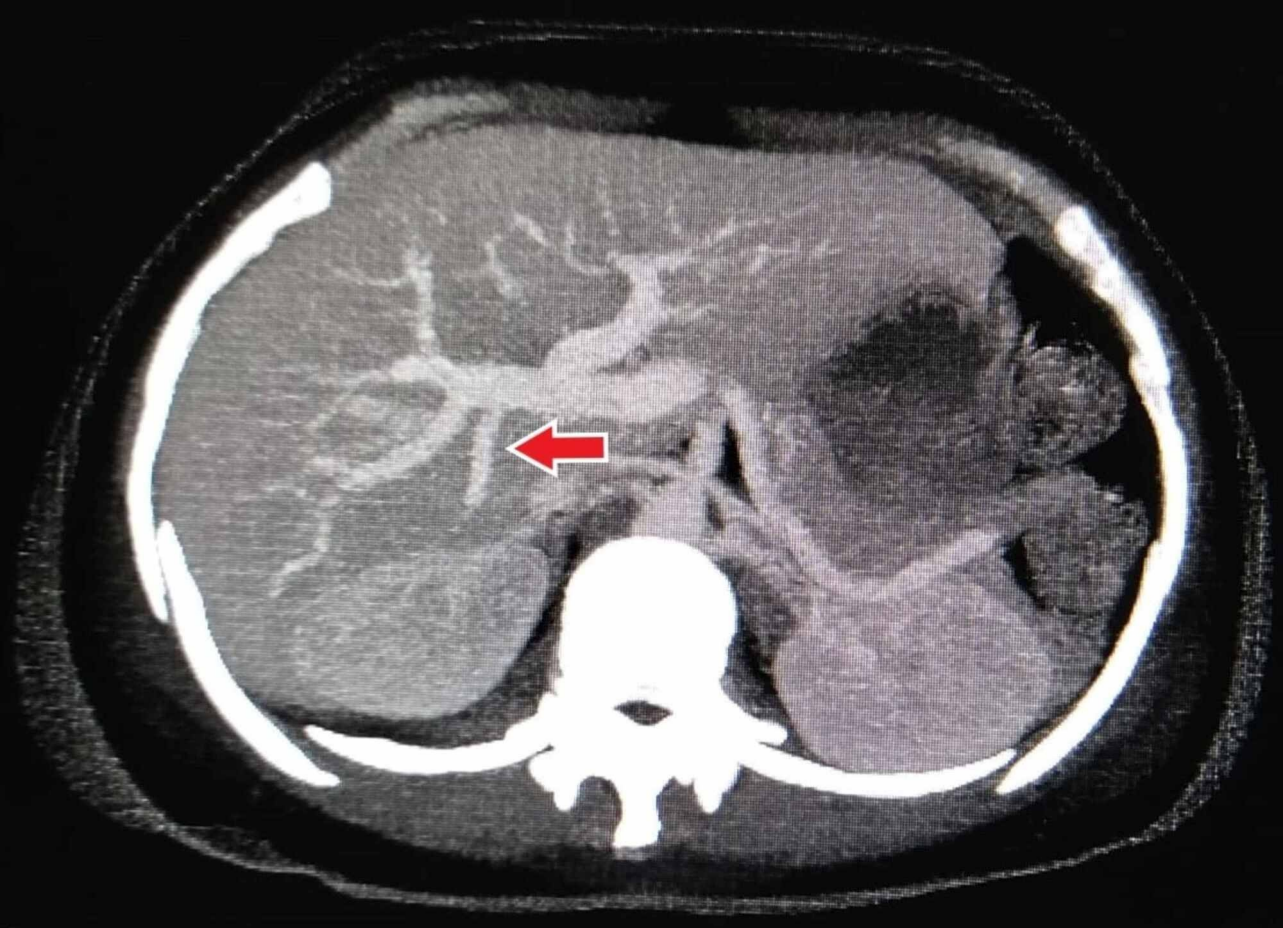
Contrast-enhanced CT MIP reconstructed coronal image showing the separate branch of the right portal vein to segment VII (type 4) MIP, maximum intensity projection.

**Figure 5:**
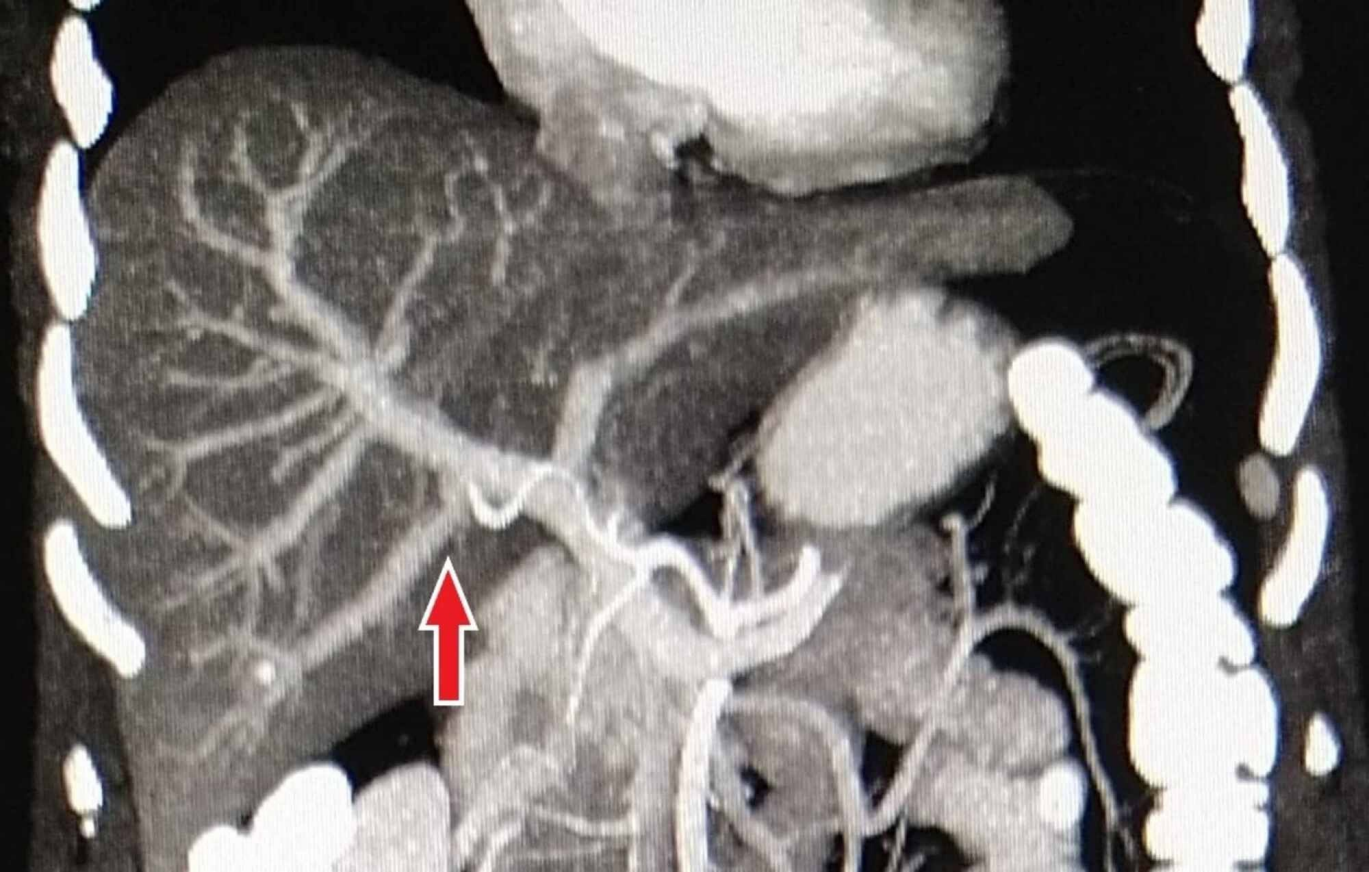
Contrast-enhanced CT MIP reconstructed coronal image showing the separate branch of the right portal vein to segment VI (type 5) MIP, maximum intensity projection.

Branch to segment VII arising separately from RPV (Figure 4) was noted in 1.2% cases, identical to frequencies described by Covey et al. and Gunasekaran and Gaba [11,13]. The supply to segment VI via the isolated branch of RPV (Figure 5) was recorded in 16 patients (3.2%). Covey et al. and Gunasekaran and Gaba also depicted type 5 as the more common pattern than type 4, corresponding to our finding [11,13].

Several other anatomical deviations have also been reported in the literature; however, these are seldom seen and pertinent data is scarce. Examples include duplications, congenital absence, undivided main portal trunk, quadrification of MPV [4], separate origin of the segment V branch from the RPV, and branching of the right anterior branch from the left portal vein [2,13]. It is important that special attention is given to portal venous anatomy in patients planned for hepatic resections and transplantations to ensure adequate graft selection and formation of suitable anastomotic channels and avoid inadvertent compromise of blood perfusion [2,13]. Moreover, the deviation in biliary system morphology is often associated with variations in portal vein and its evaluation is necessary to minimize the risk of iatrogenic insults [2,7,16].

Similarly, interventional radiology procedures like TIPS and portal vein embolization also demand precise knowledge of PV anatomy as undesirable complications such as migration of embolization material and irrepressible bleeding may occur [2,13].

In another study, Yamashita et al. emphasized upon the association of the right-sided round ligament, which is crucial for secure hepatic resections, with structural aberrations in biliary and vascular anatomy of liver [17].

In this study, we also elucidated the added advantage of MIP reconstruction in the evaluation of vascular anatomy, where 46 out of 62, i.e., 74.19% variant branching patterns (including type 2 through type 5) were identified using MIP while only 16 (25.80%) were recorded without MIP. Though this finding was not statistically significant, to our knowledge, the literature is deficient of studies demonstrating the value of MIP, and further large-scale studies evaluating the role of MIP are required.

Despite being a prospective study, the major limitation of our study is the small sample group, which is not enough to reflect PV variations in this highly populous region. Future studies should also evaluate other less common branching patterns of portal vein. Moreover, studies elaborating the association with variations in the hepatic vein and biliary system would be more helpful for dedicated transplant units.

## Conclusions

Our study displays a relatively high frequency of standard PV anatomy compared to previous studies. RPPV originating as the first branch of the main portal vein (type 3) is the most common variation seen in this study. Moreover, we conclude that MIP reconstruction improves the detection of PV variations.
